# Correction: Rivas et al. Tendon-Derived Mesenchymal Stem Cells (TDSCs) as an In Vitro Model for Virological Studies in Wild Birds. *Viruses* 2023, *15*, 1455

**DOI:** 10.3390/v15122283

**Published:** 2023-11-21

**Authors:** José Rivas, Axel Dubois, Aude Blanquer, Mazarine Gérardy, Ute Ziegler, Martin H. Groschup, Luc Grobet, Mutien-Marie Garigliany

**Affiliations:** 1 Fundamental and Applied Research for Animals & Health (FARAH), Laboratory of Pathology, Faculty of Veterinary Medicine, University of Liège, Sart Tilman B43, B-4000 Liège, Belgium; jfarivas@uliege.be (J.R.); aude.blanquer@uliege.be (A.B.); mazarine.gerardy@uliege.be (M.G.); 2 Fundamental and Applied Research for Animals & Health (FARAH), Laboratory of Embryology, Faculty of Veterinary Medicine, University of Liège, Sart Tilman B43, B-4000 Liège, Belgium; axel.dubois@uliege.be (A.D.); lgrobet@uliege.be (L.G.); 3 Institute for Novel and Emerging Infectious Diseases, Friedrich-Loeffler-Institut, Südufer 10, 17493 Greifswald-Insel Riems, Germany; ute.ziegler@fli.de (U.Z.); martin.groschup@fli.de (M.H.G.)

## Error in Table

In the original publication [1], there was an error of omission in Table 1 as published. Due to an oversight, the manuscript was published without the GenBank accession numbers being available. Given the importance of this information and that the accession numbers are now available, an update of these data are hereby requested. The corrected Table 1 appears below.

**Table 1:** Primer sequences used in the RT-PCR characterization of blackbird TDSCs.

mRNA	Accession Number	Primer	Primer Sequence	Amplicon Size (bp)
CD29	BK064246	CD29F	CATTCCCATTGTAGCCGGTG	151
		CD29R	TTCACCCGTATCCCACTTGG	
CD44	BK064237	CD44F	CCTTCTGGGTGCTGACAAAC	158
		CD44R	ATTTCCCCTGGTGTGGATCA	
CD71	BK064244	CD71F	AGATGACTCCTACTGCGTCG	200
		CD71R	GGCAGCGTTCTCATCTTCAG	
CD73	BK064243	CD73F	CCCATTGATGAGCAGAGCAC	211
		CD73R	CTGGGGCTTTGGAGAGATCA	
CD90	BK064242	CD90F	TCTCCGAGAACATCTACCGC	221
		CD90R	CCACGAGGTGTTCTGGATCA	
CD105	BK064241	CD105F	GCTGACTTCAAGGCACAACA	245
		CD105R	ATGGTGTAGGTGAAGCGGAA	
CD14	BK064239	CD14F	GTCGCCAGCTCAGTACCA	224
		CD14R	GGACACCAAGCACAGGGA	
CD34	BK064238	CD34F	GGCAGGAATTTGGGTGTGAG	233
		CD34R	TCATGTCCCTGCTCATCCTG	
CD45	BK064245	CD45F	TGACACCATTGCCAGTACCT	156
		CD45R	GTTTTCTCTGGCTGTGGTGG	
GAPDH	BK064240	GAPDH_F	TCTCTGTTGTGGACCTGACC	169
		GAPDH_R	TCAAAGGTGGAGGAATGGCT	

## Text Correction

With regard to the correction of Table 1, a correction has been made to Section 3.2, Paragraph 1.

From raw data of a Eurasian blackbird’s transcriptomic analysis [30] we deduced the mRNA sequences from the positive TDSCs markers CD29, CD44, CD71, CD73, CD90, CD105, and the negative markers CD14, CD34, and CD45. Nucleotide sequence data reported are available in the Third Party Annotation Section of the DDBJ/ENA/GenBank databases under the accession numbers TPA: BK064237-BK064246. From these mRNA sequences, we designed the primers presented in Table 1.

The authors state that the scientific conclusions are unaffected. This correction was approved by the Academic Editor. The original publication has also been updated.
